# Adaptive Behavior, Emotional/Behavioral Problems and Parental Stress in Children With Autism Spectrum Disorder

**DOI:** 10.3389/fnins.2021.751465

**Published:** 2021-11-25

**Authors:** Francesca Felicia Operto, Grazia Maria Giovanna Pastorino, Chiara Scuoppo, Chiara Padovano, Valentina Vivenzio, Ilaria Pistola, Gilda Belfiore, Rosetta Rinaldi, Valeria de Simone, Giangennaro Coppola

**Affiliations:** Child and Adolescent Neuropsychiatry Unit, Department of Medicine, Surgery and Dentistry, University of Salerno, Salerno, Italy

**Keywords:** autism spectrum disorder, adaptive functioning, emotional/behavioral problems, parental stress, neurodevelopmental disorders

## Abstract

**Background:** The aim of our study was to compare adaptive skills, emotional/behavioral problems, and parental stress among children with different severity levels of Autism Spectrum Disorder (ASD) symptoms.

**Methods:** This study included a sample of 88 subjects with ASD (mean age = 6.00 ± 2.70). All subjects underwent standardized neuropsychological tests for the assessment of symptoms of the autism spectrum (Autism Diagnostic Observation Schedule-Second Edition), adaptive level (The Vineland Adaptive Behavior Scales, Survey Interview, 2nd edition), behavioral and emotional problems (Child Behavior CheckList CBCL), and parental stress (Parental Stress Index Short Form-PSI-SF). Non-parametric statistical methods (Kruskal-Wallis test and Mann-Whitney *U*-test for *post hoc* analysis) and linear regression analysis were used in this study.

**Results:** *C*hildren who had higher severity levels of ASD symptoms had less adaptive functioning; younger children showed more severe symptoms of ASD; older children had better communication skills. The presence of greater adaptive difficulties was related to a greater presence of internalizing problems. An increase in parental stress levels was related to an higher severity of ASD symptoms, fewer adaptive skills, and a greater presence of internalizing and externalizing problems.

**Conclusion:** This study suggests that the adaptive behavior should be considered in order to planning a habilitation intervention in children with autism. It is also important to monitor emotional/behavioral problems and parental stress levels in order to provide parenting support and improve the family quality of life.

## Introduction

Autism spectrum disorder (ASD) is a neurodevelopmental disorder characterized by impaired social interaction/communication and the presence of restricted or repetitive behaviors (American Psychiatric Association [APA], 2013). Symptoms are not best explained by intellectual disability and must manifest in the early stages of development. This disorder has very heterogeneous clinical features and, according to the Diagnostic and Statistical Manual of Mental Disorders 5th edition (DSM-5), the severity of this condition ranging from mild to severe, according to three severity levels, from 1 to 3. The severity level is based on impaired social communication and restricted and repetitive patterns of behavior. In level 1, in the absence of support, social communication deficits cause significant impairment and the inflexibility of behavior causes significant interference with functioning in one or more contexts. In level 2 there are marked deficits in social communication skills and the restricted and repetitive behaviors interfere with functioning in different contexts. Level 3 is characterized by severe deficits in social communication skills and the restricted and repetitive behaviors interfere markedly with all areas of functioning (American Psychiatric Association [APA], 2013).

Research in the literature shows that in subjects with ASD there is an impairment of adaptive abilities which can increase with growth and can be present regardless of intellectual level (Kanne et al., 2011; Chatham et al., 2018; Precenzano et al., 2020; Pastorino et al., 2021).

Adaptive functioning is a construct that includes the skills necessary to live in everyday life (Matthews et al., 2015). A widely used clinical and research tool (Balboni et al., 2001; Schalock et al., 2010) to evaluate adaptive functioning is the Vineland Adaptive Behavior Scales II (VABS II; Sparrow et al., 2005). Through this structured interview, adaptive behaviors from birth up to adulthood are assessed, starting from reaching the first stages of development up to the most complex requests in the various areas. In autism studies using the Vineland II test, deficits in communication, socialization, daily living skills, and motor skills emerged (Balboni et al., 2016; Chatham et al., 2018). Difficulty in adaptive skills adversely affects the lives of individuals with ASD, impacting on daily living skills, social experiences, and school activities (Baker et al., 2008; O’Donnell et al., 2012).

In addition to adaptive difficulties, there are several studies in the literature (Havdahl et al., 2016) which show that children with ASD have a high prevalence of emotional and behavioral problems. In particular, in some researches (Ooi et al., 2010; Kempe et al., 2011) it was found that subjects with ASD had significantly higher scores than children of a mixed clinical control group in the following scales of the CBCL test: social problems, withdrawal problems/depression, attention problems, and thinking problems.

Another much investigated topic is parental stress (Operto et al., 2019a,b). The parental stress in mothers and fathers of children with ASD seem to be higher than in parents of normotypic children (Bonifacci et al., 2016; Craig et al., 2016), and this can adversely affect the general well-being of the whole family. We speak of parental stress when a parent has a psychological reaction of aversion to the request to perform their parental role and generally the parent does not perceive that there are resources available to satisfy this request (Giovagnoli et al., 2015). Many studies have shown that there are various factors that influence each other and determine parental stress, including characteristics of the parent, child, family, and ecology (Lazarus and Folkman, 1984; McCubbin, 1989). A test designed to measure various parental stressors is the Abidin Parental Stress Index (PSI) (Abidin, 1995).

Some characteristics of patients that could increase parental stress are the severity of autism spectrum symptoms, the adaptive level and emotional problems of their children, but there do not appear to be studies in the literature that have analyzed the interaction of the adaptive level with the severity of symptoms in individuals with ASD, also considering emotional/behavioral problems and parental stress level.

Therefore, the aim of our study was to evaluate adaptive skills, emotional/behavioral problems, and parental stress and compare them among children with different severity levels of ASD symptoms.

## Materials and Methods

### Participants

Our sample consisted of children and adolescents diagnosed with ASD (*n* = 88; males = 57; mean age = 6.00 ± 2.70). The participants and their parents (mother = 88; father = 88) were recruited at the Child and Adolescents Neuropsychiatry Unit—University Hospital of Salerno (Italy). The diagnosis of ASD, according to the DSM-5 criteria, were made by a team of neuropsychiatrists and psychologists, with the support of standardized and validated neuropsychological tests (Autism Diagnostic Observation Schedule Second Edition—ADOS-2 and Autism Diagnostic Interview Revised—ADI-R). The ADOS-2 modules 1–3 were used for the assessment. Patients with different levels of severity were included, calculated by means of the comparison score from 4 to 10 of ADOS-2 (Gotham et al., 2009; Fiore et al., 2020). In particular the children were divided into “low severity” level for those who obtained comparison score 4, “moderate severity” level for those with a comparison score from 5 to 7, “high severity” level for those with comparison scores of 8–10.

We also consider the following variables: age of mothers and fathers; socioeconomic status measured as educational level of the parents (in years of schooling).

The exclusion criteria were the presence of comorbidities for neurological (cerebral palsy, epilepsy, migraine), psychiatric (anxiety, depression, and psychosis), and other relevant medical conditions. All the participants performed a neuropsychological assessment using standardized tests, as in our clinical practice. All the subjects recruited agreed to participate in our study. The parents provided their written informed consent after receiving a description about the objective and the protocol of the study. The study design was approved by the Campania Sud Ethics Committee (protocol number 0144996—July, 28 2021) and it was conducted according to the rules of good clinical practice, in line with the Declaration of Helsinki.

### Measures

The evaluation of all the participants involved the following neuropsychological standardized tests:

*The Autism Diagnostic Observation Schedule Second Edition (ADOS-2)* is a standardized and semi-structured measure that assess communication, social interaction, play, and the imaginative use of materials in individuals with ASD (Lord et al., 2012). It represents the gold standard for the evaluation and diagnosis of children and young people with suspicion or diagnosis of ASD. This test consists of different Modules, according to age and language skills of the subject evaluated: Module 1 for subjects aged 31 months or older who do not have a language consistent with the sentences; Module 2 for individuals of any age who use sentence language but are not yet verbally fluent; Module 3 for verbally fluent topics. The ADOS-2 provides two separate domain categories: “Social Affect” and “Restricted, Repetitive Behaviors” from which a single “Total Score” is derived. The “Total Score” is converted into a “Comparative Score” ranging from 1 to 10. This numerical range is divided into four interpretative categories that correspond to the levels of symptoms related to the autism spectrum: levels 1 and 2 for minimum or absence of symptoms associated with the autism spectrum; 3 and 4 for a low level of symptoms; from 5 to 7 for an average level; 8 to 10 for a high level. In modules 1, 2, and 3 Cronbach’s alpha values were highest for the AS domain (0.87–0.92 for evolutionary subgroup) and ranged from 0.51 to 0.66 in the CRR domain.

*The Autism Diagnostic Interview—Revised (ADI-R)* is a semi-structured interview employed by trained examiners and used with caregivers to gather information on the development of behaviors and skills in the first years of the patient’s life (Lord et al., 1994). The interviewer codes behavioral descriptions given by the caregiver as 0 (no abnormality), 1 (possible abnormality), 2 (definite autistic type abnormality), and 3 (severe autistic type abnormality). In the present study, scores of 3 were recorded to 2, as recommended by Lord et al. (1994). Internal consistency estimates are 0.95 in the communication domain and 0.69 in the restricted and repetitive behavior domain (Lord et al., 1994). Used together, ADI-R and ADOS are considered the “gold standard” for diagnosing ASD (Falkmer et al., 2013).

*The Vineland Adaptive Behavior Scales, Survey Interview, 2nd edition (VABS)* is a parent-report questionnaire of adaptive behavior for individuals aged birth to 99 years (Sparrow et al., 2005). In this tool there is an “Adaptive Behavior Composite Score” which derives from 4 scales that, respectively, evaluate “Communication,” “Daily life skills,” “Socialization,” and “Motor skills.” These domains consist of 2 or 3 subscales each. In the single items the scores range from 0 to 2 (0 = never, 1 = sometimes, or partially, 2 = usually) through the interview with the parents, which convert into raw scores from which scores on the v-scale are obtained. Scores on the v-scale have a mean of 15 and a standard deviation of 3. Standard scales/scores were used for data analysis to allow comparison between different age groups. The motor abilities are assessed in children from birth to 6 years 11 months of age and were not considered for this study. The reliability coefficients of the Vineland subscales are between 0.80 and 0.90.

*The Child Behavior Checklist (CBCL)* is an evidence-based questionnaire (Achenbach and Rescorla, 2001) used to assess behavioral, emotional, and social problems and functioning in children up to 18 years. There are two versions compiled by the caregivers: one is used for children from 1 and a half to 5 years old and another from 6 to 18 years old. The tool consists of 113 statements and there are three possible answers recorded on a Likert scale: 0 Not true, 1 Sometimes or Fairly True, 2 Often True or Very True. The results are distributed in subscales as t-scores. The normative data are divided as follows: a t-score ≤ 64 is normal, an interval at the limits is indicated by a t-score between 65 and 69 and a t-score ≥ 70 indicates clinical symptoms. For the subscales of the “internalization problems,” “externalization problems,” and “total problems,” a t-score ≤ 59 indicates normal scores, a t-score between 60 and 64 indicates a score that is within a boundary range and high levels of maladaptive behavior are indicated by t-score ≥ 65. The CBCL have an adequate internal consistency, as emerged from the study of D’Orlando et al. (2010).

The *PSI Short Form (PSI/SF)* arises from the Parenting Complete Stress Index (PSI) test (Abidin, 1995) and is composed of 36 sentences. The parents must indicate how much they agree with each of the 36 sentences, according to a 5-point Liker scale, ranging from “strongly agree” to “strongly disagree.” In this questionnaire there are three subscales: Parental Distress (PD), which indicates the level of discomfort that a caregiver is experiencing as a parent, also taking into consideration personal factors directly related to that role, Dysfunctional Parent-Child Interaction Scale (P-CDI), which assesses the level of satisfaction linked to the relationship with the child, and finally Difficult Child Scale (DC) which assesses the parent’s perception of having a difficult child (Abidin, 2012). In PSI/SF the raw score is converted to t-scores; the higher the t-scores the higher the stress levels, in particular a t-score ≥ 85 indicates clinically significant parental stress (Abidin, 1995). The test also includes a defensive response scale (DF) useful for verifying the validity of the protocol as it indicates whether the parent tends, for example, to give a better self-image or to minimize problems and perceived stress in the relationship the child.

## Statistical Analysis

All the measures were expressed as mean and standard deviation (SD). The mean score of ADOS-2, VABS-II, PSI, and CBCL was compared among ASD groups (levels low-moderate-high) using the non-parametric Kruskal-Wallis test and with the subsequent *post hoc* analysis using the Mann-Whitney *U*-test. The comparison between the age groups, based on the two versions of the CBCL (<6 years; ≥ 6 years), and sex groups, was made using Mann–Whitney *U*-test.

The linear regression analysis was performed in order to evaluate the relationships between different variables (age, ADOS-2 scores, VABS-II scores, CBCL scores, and PSI scores). The strength of relationship ranging from 1 to 1, with −1 indicating a perfect negative linear relation, 1 indicating a perfect positive linear relation, and 0 indicating no linear relation between variables. A *p*-value of less than 0.05 was considered as statistically significant. For statistical processing, we used the data processing program the Statistical Package for Social Science version 23.0.

## Results

The three group based on severity level of ASD symptoms did not significantly differ in all the main socio-demographic characteristics (Table 1).

**TABLE 1:** Sample characteristics.

	Total ASD *n* = 88	Level low *n* = 28	Level moderate *n* = 34	Level high *n* = 26	Statistics
Male	57 (65%)	19 (68%)	23 (68%)	15 (58%)	Chi square test χ^2^ = 0.8076; *p* = 0.6677
Female	31 (35%)	9 (32%)	11 (32%)	11 (42%)	
Age in years (M ± SD)	6.00 ± 2.70	7.07 ± 3.07	5.62 ± 2.51	5.35 ± 2.24	Kruskall-Wallis *U*-test χ^2^ = 5.558; *p* = 0.063
Mother age in years (M ± SD)	32.48 ± 3.51	33.21 ± 4.02	32.50 ± 3.41	31.71 ± 3.12	Kruskall-Wallis *U*-test χ^2^ = 0.960; *p* = 0.619
Father age in years (M ± SD)	34.93 ± 3.99	35.07 ± 4.51	35.14 ± 3.23	34.57 ± 4.38	Kruskall-Wallis *U*-test χ^2^ = 0.053; *p* = 0.974
Maternal educational level* (M ± SD) *n* = 88	12.40 ± 3.39 *n* = 88	12.43 ± 3.32 *n* = 28	11.79 ± 3.24 *n* = 34	13.00 ± 3.72 *n* = 26	Kruskall-Wallis *U*-test χ^2^ = 2.298; *p* = 0.317
Paternal educational level* (M ± SD) *n* = 62	12.33 ± 3.16 *n* = 62	11.93 ± 2.13 *n* = 19	11.71 ± 3.67 *n* = 24	13.36 ± 3.43 *n* = 19	Kruskall-Wallis *U*-test χ^2^ = 2.339; *p* = 0.310

*ASD, Autism Spectrum Disorder; M, mean; SD, Standard Deviation.*

*Expressed in age of schooling.

The analysis conducted to compare VABS, PSI, and CBCL mean scores among ASD levels showed a significant difference in the three following scales of VABS questionnaire: Communication, Daily Living Skills, Socialization and Adaptive Behavior Composite. No other differences were found. Average scores and statistical analysis were reported in Table 2.

**TABLE 2:** Mean scores comparison among the three ASD severity levels.

	ASD Level low *n* = 28	ASD Level moderate *n* = 34	ASD Level high *n* = 26	Statistic Kruskall-Wallis test
VABS_COM (M ± SD)	78.3716.03	55.7318.04	44.2310.85	**χ^2^ = 39.357; *p* = < 0.05**
VABS_DLS (M ± SD)	79.1515.14	64.1817.63	54.969.82	**χ^2^ = 28.089; *p* = < 0.05**
VABS_SOC (M ± SD)	73.4413.73	61.5215.38	55.6910.17	**χ^2^ = 22.234; *p* = < 0.05**
VABS_ABC (M ± SD)	74.6314.22	56.3319.75	47.6314.50	**χ^2^ = 29.092; *p* = < 0.05**
PSI_PD (M ± SD)	72.8819.30	59.0328.48	71.0023.50	χ^2^ = 4.034; *p* = 0.133
PSI_PCDI (M ± SD)	66.3518.45	76.5520.18	77.6816.51	χ^2^ = 5.356; *p* = 0.069
PSI_DC (M ± SD)	69.8121.28	69.3124.92	71.0018.43	χ^2^ = 0.089; *p* = 0.956
PSI_TS (M ± SD)	71.3518.34	67.7626.34	79.2016.75	χ^2^ = 3.282; *p* = 0.198
CBCL_INT (M ± SD)	55.7210.38	57.8810.25	56.9210.00	χ^2^ = 0.505; *p* = 0.777
CBCL_EXT (M ± SD)	53.4410.52	54.259.48	52.529.34	χ^2^ = 0.472; *p* = 0.790
CBCL_TP (M ± SD)	56.1611.89	57.5311.01	57.009.48	χ^2^ = 0.023; *p* = 0.989

*M, mean; SD, Standard Deviation; VABS_COM, Vabs Communication; VABS_DLS, Vabs Daily Living Skills; VABS_SOC, Vabs Socialization; VABS_ABC, Vabs Adaptive Behavior Composite; PSI_PD, parental distress; PSI_PCDI, dysfunctional interaction parent-child; PSI_TS, Total Stress; PSI_DC, difficult child; CBCL_INT, cbcl internalization problems; CBCL_EXT, cbcl externalization problems; CBCL_TP, cbcl total problems.*

*p < 0.05 are in bold.*

*Post hoc* analysis revealed that children ASD level low had a significant higher score in all subscales of VABS than children ASD level moderate and level high; in addition, subjects ASD level moderate showed higher significant score than subjects ASD level high in all the VABS subscales, except Socialization scale. *Post hoc* analysis was reported in Table 3.

**TABLE 3:** *Post Hoc* analysis (Mann-Whitney *U*-test).

	VABS_COM	VABS_DLS	VABS_SOC	VABS_ABC
**1 vs. 2**	***U* = 122.00 *p* < 0.001**	***U* = 234.50 *p* = 0.002**	***U* = 237.50 *p* = 0.002**	***U* = 182.50 *p* < 0.001**
**1 vs. 3**	***U* = 28.00 *p* < 0.001**	***U* = 51.50 *p* < 0.001**	***U* = 93.00 *p* < 0.001**	***U* = 71.50 *p* < 0.001**
**2 vs. 3**	***U* = 258.00 *p* = 0.009**	***U* = 280.00 *p* = 0.023**	*U* = 306.00 *p* = 0.060	***U* = 289.00 *p* = 0.032**

*1, low level group; 2, moderate level group; 3 high level group, VABS_COM, Vabs Communication; VABS_DLS, Vabs Daily Living Skills; VABS_SOC, Vabs Socialization; VABS_ABC, Vabs Adaptive Behavior Composite.*

*Significant p-values are in bold.*

The comparison between Age groups among ADOS, VABS, PSI, and CBCL scores showed that the ADOS Social Affect and ADOS Total Score of group Age < 6 was significantly higher than group Age ≥ 6, while in VABS Communication and in PSI Difficult Child and PSI Parental Distress the group Age ≥ 6 exhibited significant higher scores than group Age < 6. The mean scores and statistical analysis were reported in Table 4. The comparison between Sex groups among ADOS, VABS, PSI, and CBCL didn’t show statistical differences.

**TABLE 4:** Mean scores comparison between Age groups.

	Age < 6 (*n* = 41)	Age ≤ 6 (*n* = 47)	Mann– Whitney *U*-test
ADOS_AS (M ± SD)	12.384.62	9.833.92	***U* = 630.00; *p* = 0.008**
ADOS_RRB (M ± SD)	3.481.26	3.171.52	*U* = 821.00; *p* = 0.298
ADOS_TOT (M ± SD)	15.855.04	13.004.93	***U* = 637.00; *p* = 0.010**
ADOS_Comp (M ± SD)	6.101.37	6.151.69	*U* = 909.00; *p* = 0.947
VABS_COM (M ± SD)	54.2816.66	63.7822.82	***U* = 677.00; *p* = 0.035**
VABS_DLS (M ± SD)	66.3514.37	65.8720.11	*U* = 896.00; *p* = 0.835
VABS_SOC (M ± SD)	64.4813.46	62.6516.53	*U* = 874.50; *p* = 0.693
VABS_ABC (M ± SD)	61.9018.41	57.3320.81	*U* = 800.50; *p* = 0.301
PSI_PD (M ± SD)	59.4627.05	74.0020.62	***U* = 541.00; *p* = 0.014**
PSI_PCDI (M ± SD)	72.0317.46	74.9320.34	*U* = 695.00; *p* = 0.329
PSI_DC (M ± SD)	63.6422.19	75.4719.75	***U* = 544.00; *p* = 0.015**
PSI_TS (M ± SD)	68.9223.40	75.5816.62	*U* = 663.00; *p* = 0.119
CBCL_INT (M ± SD)	56.5110.00	57.2710.33	*U* = 822.00; *p* = 0.922
CBCL_EXT (M ± SD)	52.358.85	54.4010.30	*U* = 766.50; *p* = 0.538
CBCL_TP (M ± SD)	55.279.43	58.3311.62	*U* = 694.50; *p* = 0.198

*ADOS_SA, Social Affect; ADOS_RRB, Restricted and Repetitive Behaviors; ADOS_TOT, Ados Total Score; ADOS_Comp, Comparison Scores; VABS_COM, Vabs Communication; VABS_DLS, Vabs Daily Living Skills; VABS_SOC, Vabs Socialization; VABS_ABC, Vabs Adaptive Behavior Composite; PSI_PD, parental distress; PSI_PCDI, dysfunctional interaction parent-child; PSI_DC, difficult child; PSI_TS, Total Stress; CBCL_INT, cbcl internalization problems; CBCL_EXT, cbcl externalization problems; CBCL_TP, cbcl total problems.*

*p < 0.05 are in bold.*

### Linear Regression Analysis

Linear regression analysis revealed a significant relationship between several factors (Table 5).

**TABLE 5:** Linear regression analysis.

		ADOS_SA	ADOS_RRB	ADOS_TOT	ADOS_COMP	VABS_COM	VABS_DLS	VABS_SOC	VABS_ABC	PSI_PD	PSI_PCDI	PSI_DC	PSI_TOT	CBLB_INT	CBCL_EXT	CBCL_TP
Age	F	4.870	0.193	4.029	1.943	10.799	0.218	2.519	1.094	2.333	1.278	7.894	1.279	0.623	0.117	1.028
	Beta	–0.223	–0.048	–0.213	0.150	0.338	–0.051	–0.171	–0.113	0.170	0.127	0.303	0.127	0.088	0.038	0.113
	*p*	0.030*	0.662	0.048*	0.167	0.001*	0.642	0.116	0.299	0.131	0.262	0.006*	0.262	0.432	0.734	0.314
ADOS_SA	*F*					41.784	18.234	21.486	21.590	0.019	4.003	0.298	1.772	2.232	0.201	1.461
	Beta					–0.576	–0.422	–0.451	–0.361	0.016	0.221	0.062	0.149	0.165	0.050	0.134
	*p*					0.000*	0.000*	0.000*	0.001*	0.889	0.049*	0.587	0.187	0.139	0.655	0.230
ADOS_RRB	*F*					17.552	12.008	9.002	10.654	3.316	1.202	0.922	0.164	0.015	0.248	0.212
	Beta					–0.416	–0.354	–0.311	–0.335	–0.202	0.123	0.108	–0.046	0.014	0.056	0.051
	*p*					0.000*	0.001*	0.004*	0.002*	0.72	0.276	0.340	0.686	0.901	0.620	0.646
ADOS_TOT	*F*					49.610	22.520	24.241	16.197	0.130	4.108	0.532	1.067	1.704	0.272	1.346
	Beta					–0.609	–0.460	–0.473	–0.402	–0.041	0.224	0.082	0.116	0.144	0.058	0.129
	*p*					0.000*	0.000*	0.000*	0.000*	0.719	0.046*	0.468	0.305	0.195	0.603	0.249
ADOS_Comp	*F*					7.831	7.936	16.470	6.128	0.180	1.433	3.031	0.173	3.723	1.474	4.142
	Beta					–0.294	–0.295	–0.407	–0.262	–0.048	0.135	0.195	0.047	0.212	0.135	0.223
	*p*					0.006*	0.006*	0.000*	0.015*	0.673	0.235	0.086	0.678	0.057	0.228	0.045*
VABS _COM	*F*									0.214	5.321	0.043	2.349	2.198	1.308	2.108
	Beta									0.052	–0.253	–0.023	–0.171	–0.164	–0.127	–0.160
	*p*									0.645	0.024*	0.836	0.129	0.142	0.256	0.150
VABS _DLS	*F*									0.185	2.403	1.698	2.488	2.730	1.183	2.363
	Beta									–0.049	–0.173	–0.146	–0.176	–0.182	–0.121	–0.169
	*p*									0.668	0.125	0.196	0.199	0.102	0.280	0.128
VABS _SOC	*F*									0.065	6.399	1.941	1.976	4.452	0.618	2.258
	Beta									–0.029	–0.275	–0.156	–0.157	–0.230	–0.088	–0.166
	*p*									0.799	0.013*	0.168	0.164	0.038*	0.434	0.137
VABS _ABC	*F*									0.411	5.204	1.577	3.604	1.228	0.569	1.446
	Beta									–0.072	–0.250	–0.141	–0.210	–0.123	–0.084	–0.133
	*p*									0.532	0.025*	0.213	0.061	0.271	0.453	0.233
PSI_PD	*F*													2.577	1.939	2.923
	Beta													0.181	0.158	0.192
	*p*													0.113	0.168	0.091
PSI_PCDI	*F*													10.214	3.373	8.221
	Beta													0.344	0.297	0.312
	*p*													0.002*	0.008*	0.005*
PSI_DC	*F*													11.297	14.346	12.769
	Beta													0.360	0.398	0.379
	*p*													0.001*	0.000*	0.001*
PSI_TS	*F*													8.251	10.609	11.201
	Beta													0.313	0.350	0.358
	*p*													0.005*	0.002*	0.001*

*ADOS_SA, Social Affect; ADOS_RRB, Restricted and Repetitive Behaviors; ADOS_TOT, Ados Total Score; ADOS_Comp, Comparison Scores; VABS_COM, Vabs Communication; VABS_DLS, Vabs Daily Living Skills; VABS_SOC, Vabs Socialization; VABS_ABC, Vabs Adaptive Behavior Composite; PSI_PD, parental distress; PSI_PCDI, dysfunctional interaction parent-child; PSI_DC, difficult child; PSI_TS, Total Stress; CBCL_INT, cbcl internalization problems; CBCL_EXT, cbcl externalization problems; CBCL_TP, cbcl total problems.*

*Significant p-value.

We found negative relationship between Age and ADOS Social Affect, ADOS Total Score, and positive relationship among Age and VABS Communication, and PSI Difficult Child.

All the ADOS scores correlate negatively with all VABS subscale scores. In addition we found positive relationship among ADOS Social Affect and ADOS Total Score with PSI Dysfunctional Parent-Child Interaction. Moreover ADOS Comparison scores was positively related with CBCL Total problems.

Negative relationship was found between VABS Communication, VABS Socialization, VABS Adaptive Behavior Composite, and PSI Dysfunctional Parent-Child Interaction; furthermore VABS Socialization and CBCL Internalizing problems was negatively related.

The CBCL Internalizing problems, CBCL Externalizing problems, and CBCL Total problems scores correlate positively with PSI Dysfunctional Parent-Child Interaction, PSI Difficult Child and PSI Total Stress. No other relationships were found. All the results are summarized in Table 5.

## Discussion

The goal of this study was to compare adaptive skills, emotional/behavioral problems, and parental stress among children with different severity levels of ASD. The results of this study may help the clinician to delineate a more specific neuropsychological profile of children with ASD and helping to further differentiate some specific characteristics of ASD individuals with different severity level of symptoms. Furthermore, knowing the adaptive functioning of children with ASD can be useful for planning targeted interventions and for assessing adaptive abilities over time, considering their strengths and weaknesses.

The first result that emerged from the analysis among the three groups, was that a higher level of ASD symptoms was correlated to less adaptive skills. In fact, the performance obtained in all VABS subscales is higher in children with level low of ASD symptoms than in those with moderate and high level. Our finding agreed with a previous study (Tillmann et al., 2019) in which the authors showed that higher levels of ASD symptoms, specifically social communication symptoms, were associated with lower adaptive functioning. Furthermore, children with level moderate ASD obtained better results in all VABS subscales than those of level high, except in Socialization, in which the two groups do not significantly differed. This finding agrees with previous studies (Yang et al., 2016; Chatham et al., 2018) in which the socialization skills were the most compromised domain in individuals with autism; again, autism severity was negatively correlated with the adaptive behavior in daily living, communication, and global adaptive functioning score (Yang et al., 2016).

Another finding that emerged from our study was that older ASD subjects (>6 years) had better communication skills than younger children (<6 years), as shown by the higher score in the VABS communication scale.

From linear regression analysis, we found a negative relationship between age and ASD symptoms. Indeed, in our sample younger children showed higher level of ASD symptoms, especially in social and communicative skills (measured by ADOS Social Affect and ADOS Total Score scales). This result is in line with the study by Yang et al. (2016), who reported a negative correlation between age and the ADOS severity score.

We also found a positive relationship between age and adaptive skills, particularly in communication competences (VABS Communication). This result could suggest that children and adolescents with ASD can obtain an improvement in some aspects of their communication skills over time, probably also due to the rehabilitation interventions, while the difficulties in socialization skills and the daily living skills persist. The study by Tillmann et al. (2019), on 417 participants aged 6–31 years, showed that older age was associated with lower adaptive functioning in all VABS subscales. The discrepancy with our study is probably due to the different range of age considered.

Parental Stress related to the child characteristics (PSI Difficult Child) were positively related to the age of child, so the parents of older children reported higher stress levels. This data underlines that parents perceive greater difficulties in family management with the increasing age of their children.

The ASD symptoms were negatively related to adaptive functioning. As showed in our analysis, children with higher severity of ASD symptoms (ADOS Social Affect, ADOS Restricted, and Repetitive Behavior, ADOS Total Score) had fewer communicative, social, and daily living skills (VABS Communication, VABS Daily Living Skills, VABS Socialization, VABS Adaptive Behavior Composite). Our results are in agreement with several previous literature studies, in which it was reported that greater communication-relational impairment was associated with greater difficulties in adaptive skills (Yang et al., 2016; Chatham et al., 2018; Tillmann et al., 2019).

The presence of higher ASD symptom (ADOS Social Affect and ADOS Total Score) was related to higher Parental Stress, generating difficulties in the parent-child relationship (PSI Dysfunctional Parent-Child Interaction). Also low adaptive skills in socialization and communication (VABS Communication, VABS Socialization, VABS Adaptive Behavior Composite) generated higher levels of parental stress (PSI Dysfunctional Parent-Child Interaction). Several chronic conditions often generate increased parental stress (Craig et al., 2016; Operto et al., 2019a,b). Neurodevelopmental disorders are associated with an increase in parental concerns about the child’s clinical condition, core symptoms, comorbidities, the child’s integration into social contexts, future independence (Operto et al., 2021).

Higher severity of ASD symptoms (ADOS Comparison scores) was also associated to more emotional and behavioral problems in children (CBCL Total problems). The difficulties in adapt to the social context (VABS Socialization) were also related to more internalizing problems in children (CBCL Internalizing Problems). Internalizing and externalizing problems are very common in children and adolescents with ASD, such as anxiety, depression, attention problems, behavioral problems (Craig et al., 2016; Guerrera et al., 2019; Operto et al., 2021). However, to the best of our knowledge there is no previous work analyzing the emotional and behavioral profile in relation to adaptive skills.

Finally, another important result of our study was that the higher presence of internalizing and externalizing problems in children (CBCL Internalizing problems, CBCL Externalizing problems, and CBCL Total problems) were positively related with higher level of parental stress, leading to the perception of having a difficult child and a complicated parent-child interaction (PSI Difficult Child, PSI Dysfunctional Parent-Child Interaction, and PSI Total Stress). Our results agree with recent studies (Operto et al., 2021) in which the parents of these children experience high levels of stress in their parental role and have the perception of having a difficult child; this could be due to the parents’ difficulty in obtaining the child’s cooperation or managing her behavior.

The main limitation of our work is the cross-sectional design that does not give information about causal relationship between the tested variables. Future prospective studies will be important to test statistical and clinical relevance of the selected variables. Furthermore, another weaknesses of the study are that, although PSI and CBCL have good psychometric properties and are important for assessing internalizing states, they are self-correlated subjective measures that could lead to possible bias.

One of the main strengths of our study is the sample size. In addition, another important strength is that our results further contribute to underline the importance of an even more patient-centered approach, that practitioners and clinicians could try to adopt when working with ASD children. As already showed in a previous review of Weitlauf et al. (2014), our study also suggests the need to evaluate different clinical aspects such as adaptive skills or emotional and behavioral features, that may differ among the ASD subjects in the same ASD severity level, contributing to personalized interventions.

The results of our study could help to better understand some specific characteristics of the different severity levels of ASD, highlighting the strengths, and weaknesses of the each individual subject and proposing more targeted treatments. Our study suggests to emphasize an individualized treatment that, in addition to the ASD symptoms severity level, also take into account the adaptive level (personal, social, and daily life skills) and the emotional and behavioral problems of the children. In fact, identifying and treating early these children’s weaknesses, we could help to improve their quality of life and prevent emotional-behavioral symptoms. Furthermore, given the level of stress that emerged in the parents of children with ASD, the importance of extending the intervention to the whole family to favor a better development and adaptation of the child must be considered.

## Data Availability Statement

The raw data supporting the conclusions of this article will be made available by the authors, without undue reservation.

## Ethics Statement

The studies involving human participants were reviewed and approved by the Campania Sud Ethics Committee. Written informed consent to participate in this study was provided by the participants’ legal guardian/next of kin.

## Author Contributions

FO conceptualized the work. CS, CP, and VV performed psychometric measurements and analyzed the data. IP, GB, and VS drafted the manuscript and revised the language. RR researched the data in the literature. GP analyzed the data, drafted the manuscript, involved in planning, and supervised the work. GC was involved in planning and supervised the work. All authors have agreed to this final version and participated in a meaningful way in the preparation of the manuscript.

## Conflict of Interest

The authors declare that the research was conducted in the absence of any commercial or financial relationships that could be construed as a potential conflict of interest.

## Publisher’s Note

All claims expressed in this article are solely those of the authors and do not necessarily represent those of their affiliated organizations, or those of the publisher, the editors and the reviewers. Any product that may be evaluated in this article, or claim that may be made by its manufacturer, is not guaranteed or endorsed by the publisher.
